# The effect of an organic rumen-protected fat supplement on performance, metabolic status, and health of dairy cows

**DOI:** 10.1186/s12917-019-2199-8

**Published:** 2019-12-11

**Authors:** Diego Manriquez, Liang Chen, Pedro Melendez, Pablo Pinedo

**Affiliations:** 1 Department of Animal Sciences, Colorado State University, Fort Collins, CO 80523 USA; 2 Aurora Organic Dairy, Platteville, Colorado 80651-9008 USA; 3 Department of Population Health, College of Veterinary Medicine, University of Georgia, Athens, GA 30602 USA

**Keywords:** Fat, Transition, Rumen, Fatty acids, Organic

## Abstract

**Background:**

Negative energy balance during the transition period is a concern for both conventional and certified organic dairy systems. During early lactation, supplemental strategies are used to cope with nutrient deficiencies that are associated with impaired health, production, and reproduction. As organic certified dairies in the United States face restricted access to dietary supplements, the evaluation of products especially formulated for organic production is needed. Our objective was to assess the effect of supplementing 0.45 kg/d of an organic rumen-protected fat (RPF) on health, metabolic status, and productive and reproductive performance of organic certified Holstein cows from 1 to 150 days in milk (DIM). Two-hundred and two cows were enrolled in a randomized blocked controlled trial conducted from January to July 2017 in a certified organic dairy located in Northern Colorado (USA). Two groups were randomly assigned to be individually supplemented with organic RPF (ORG; *n* = 100) or control pellets (CON; *n* = 102) once per day, in addition to the total mixed ration (TMR). Outcomes of interest included milk yield (kg/d) and milk components, serum concentration of glucose, and non-esterified fatty acids (NEFA), resumption of cyclicity, time-to-first artificial insemination (AI), conception at first AI, and conception within 150 DIM, disease occurrence, culling, mortality.

**Results:**

A significant effect for the inclusion of RPF was found in daily milk yield; RPF supplemented cows had greater milk yield (1.6 kg/d) compared to CON cows up to 150 DIM (*P* = 0.03). During grazing, multiparous (MP) ORG cows had greater milk yield compared to MP CON cows, whereas no effect was found in primiparous (PP) cows. Health outcomes, serum metabolite concentrations, and reproductive performance were not affected by the inclusion of RPF. Body condition loss was smaller in the ORG group up to 80 DIM; however, there was no effect on body condition during the grazing season and in the overall study period.

**Conclusions:**

These results indicate that supplementation of RPF increased daily milk yield and prevented body condition loss during at 90 DIM. However, RPF supplementation did not affect health, serum metabolite concentration, milk components, and reproductive outcomes.

## Background

Transition cows are challenged by nutrient deficits to support milk production, which triggers mobilization of fat, labile protein, and calcium [1]. These metabolic changes, combined with suboptimal dry matter intake (DMI), increase the risk of concomitant health disorders that occur disproportionately during transition [2, 3]. Health disorders with high incidence during this period include milk fever (5 to 7% [4];), subclinical ketosis (22.4 to 55.7% [5];), retained fetal membranes (4%, after a normal calving [6];), metritis (18.5 to 27.6% [7];) and displaced abomasum (3.5% [8];), besides increased severity of mastitis [9]. All of these disorders have adverse effects on animal welfare, milk production, reproduction, and farm profitability [10, 11].

Nutritional management of transition cows is commonly reported as a preventative strategy [4, 12, 13] to maintain an adequate health status through a holistic view of the cow’s metabolism. Actions include supporting the energy, protein, and calcium requirements, as well as favoring the immune and rumen function [1] by providing readily usable sources of nutrients within 21 days after calving. However, the lower energy content of nonstructural carbohydrates compared to fats and a higher risk of rumen acidosis represents a challenge to satisfy the ruminal fermentation that leads to adequate volatile fatty acids (VFA) synthesis and lactogenesis. Therefore, increasing the energy density per gram of ration with a less rumen acidogenic diet becomes an important opportunity to reduce the magnitude of the negative energy balance (NEB) in this period [14–16].

Fats are energetically denser than carbohydrates [17]. Nonetheless, extensive research has shown that, even at low levels of supplementation, fats decrease the DMI, depress ruminal fiber digestion, and are likely to produce fatty acid isomers that cause milk fat depression [18]. On the other hand, rumen-protected fats (RPF) are fatty acids (FA) combined with calcium salts that circumvent rumen fermentation and increase their availability in the small intestine [18, 19]. Therefore, RPF could represent an alternative to increase dietary energy density for transition dairy cows, without affecting the ruminal function [20].

Most commercial RPF contain calcium soaps of palmitic and oleic FA [21], with suggested supplementing amounts ranging from 0.4 to 1.5 kg/d per head. Although there are few studies published on the use of RPF during transition, the evidence suggests that RPF supplementation increases milk yield and reproductive performance [20, 22, 23]. This information has captured the attention of US organic dairy farmers; however, most commercial RPF are not permitted in certified organic dairy farms [24]. Recently, an organic RPF (Organilac®, Organic Animal Nutrition, Boulder, CO, USA), containing palm oil and whey protein, has been approved for use in certified organic dairies in the US. However, the effect of this organic RPF has not been tested in controlled feeding trials.

In this study, we hypothesized that the supplementation of an organic RPF, in a form of treatment pellets (ORG group), will improve productive performance, metabolic status, reproductive performance, and health when compared to control cows (CON group) fed with a control pellet elaborated to match all ingredients except the RPF. Thus, the general aim of this study was to assess the effect of supplementing 0.45 Kg/d of an organic RPF from 1 to 150 DIM on health and metabolic, productive and reproductive status of lactating organic Holstein cows.

## Results

### Productive performance

**Fig. 1:**
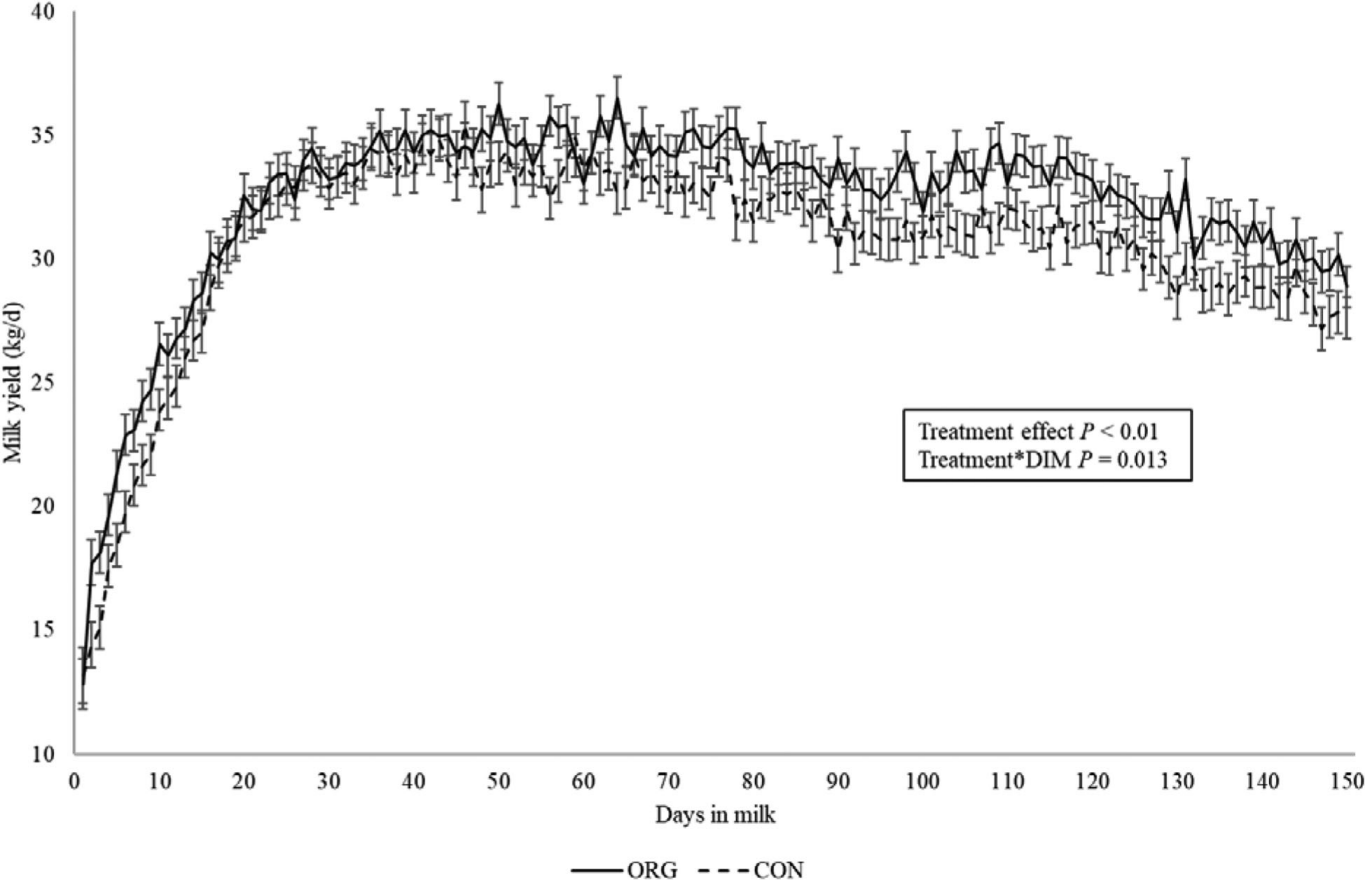
Study period milk yield from rumen protected fat (ORG) and control (CON) groups. Legend: Daily Milk yield (kg/d) least square means and standard error bars of the treatment groups from 1 up to 150 DIM. Treatment effect *P* = 0.01, interaction treatment by days in milk *P* = 0.013

Milk yield (kg/d) was evaluated until 150 DIM, as well as during grazing season and by 305 DIM. Cows in the ORG group produced 1.6 kg/d more milk compared to CON cows (32.2 ± 0.56 vs. 30.5 ± 0.55 kg/d; *P* = 0.03) up to 150 DIM. Additionally, parity and DIM (*P* < 0.0001) and the interaction term between treatment group and DIM had significant effects (*P* = 0.015). Milk yield by study group across time can be observed in Fig. 1, where the most remarkable differences occurred during the first 30 DIM and during grazing.

**Fig. 2:**
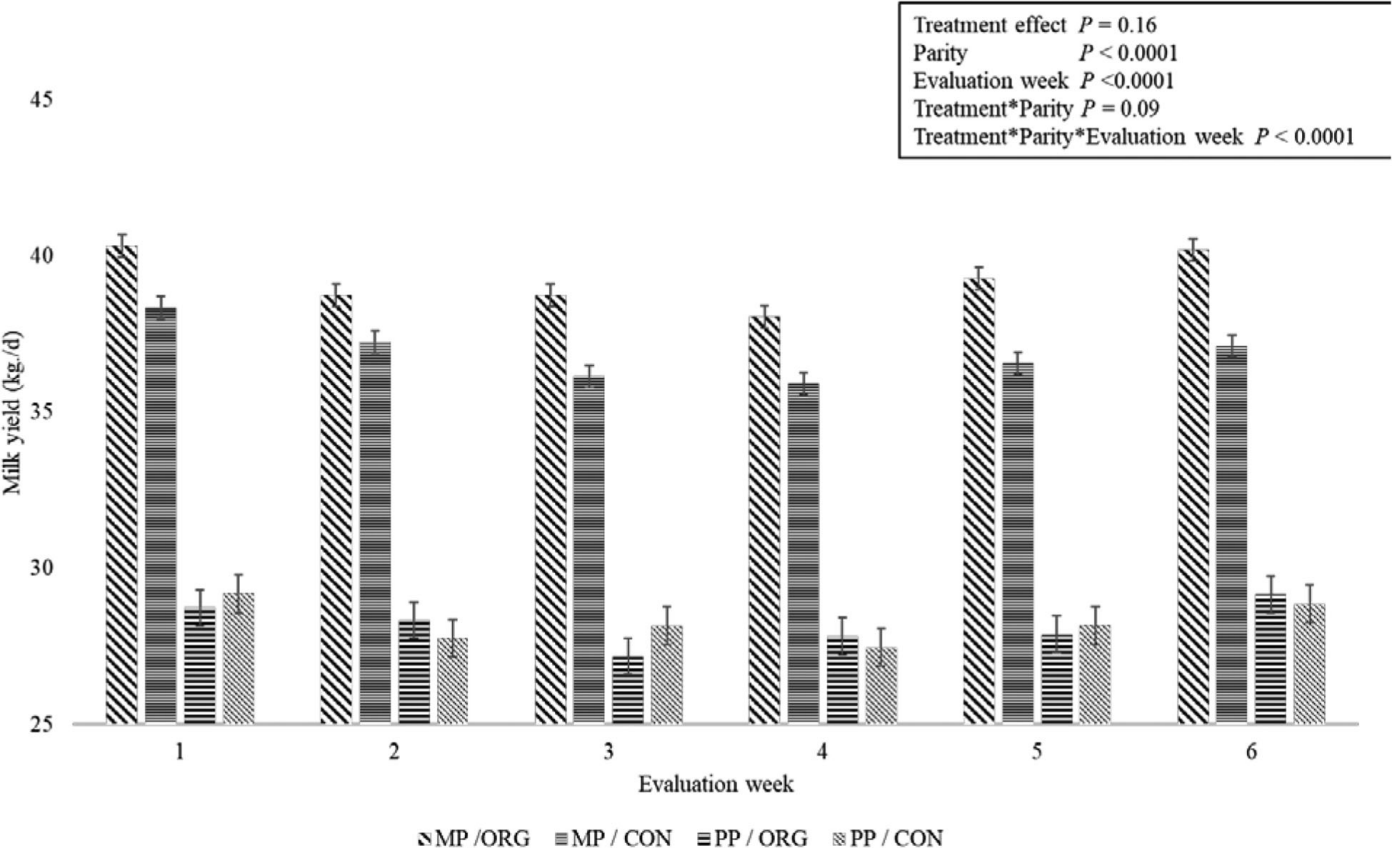
Weekly milk yield from rumen protected fat (ORG) and control (CON) groups during grazing season. Legend: Weekly milk yield (kg/d) least square means and standard error bars of the treatment groups of cows during the week prior (− 1) and during the grazing season. Parity effects = MP: multiparous; PP: primiparous. *P*-values of the main effects are provided in the textbox. *P*-values symbols: * < 0.05, ** < 0.01, *** < 0.001

A weekly analysis of milk yield was performed during the grazing season. Both treatment groups started the grazing season at around 93 DIM (ORG: 93.5 ± 0.66 vs. CON: 93.5 ± 0.65; *P* = 0.96). Average daily milk yield for the 7-d before grazing was considered as baseline. Overall, MP ORG cows receiving the rumen-protected fat produced more milk the week before the start of grazing, compared to MP CON cows (40.3 ± 0.35 vs. 38.3 ± 0.36 kg/d; *P* = 0.02). During the first week of grazing no differences were observed between ORG and CON among MP cows (38.7 ± 0.35 vs. 37.22 ± 0.36 kg/d; *P* = 0.33). In contrast, in the following weeks MP ORG cows had greater milk yield compared to MP CON cows. However, these differences were not observed among PP cows (Fig. 2).

Fat, protein, and 3.5% fat corrected milk (FCM) were compared from the farm test day performed every 15 days during the whole study period. Milk components were tested for a total of seven times. However, depending on the calving dates, there was some variation in DIM among the study cows at the time of each testing (26 d difference between the first and last enrolled cow). For this reason, we controlled the analysis of milk components by DIM. A summary of milk components by study group is presented in Table 1. No differences between treatment groups were established in the number of cows sampled per evaluation time (*P* = 0.9). As depicted in Table 1, there were no differences in milk fat, protein or FCM between study groups.

**Table 1:** Comparison of milk components between rumen protected fat (ORG) and control (CON) groups

Evaluation time	Variable	ORG	CON	Difference	*P*-value
1	Fat (%)	4.37	4.39	−0.02	0.99
	Protein (%)	2.96	3.03	−0.07	0.53
	FCM (kg)	43.7	43.6	0.1	0.99
2	Fat (%)	3.84	3.88	−0.04	0.99
	Protein (%)	2.62	2.63	−0.01	0.99
	FCM (kg)	43.1	43.3	−0.2	0.99
3	Fat (%)	3.61	3.68	−0.07	0.99
	Protein (%)	2.64	2.62	0.02	0.99
	FCM (kg)	41	41.4	−0.4	0.99
4	Fat (%)	3.63	3.73	−0.1	0.93
	Protein (%)	2.72	2.7	0.02	0.97
	FCM (kg)	41.5	41.4	0.1	0.99

Evaluation time 1: samples collected between 1 and 30 DIM; Evaluation time 2: samples collected between 31 and 50 DIM; Evaluation time 3: samples collected form 51–100 DIM; Evaluation time 2: samples collected between 101 and 150 DIM

Finally, daily milk yield (kg/d) for the overall 305 d period was compared between treatment groups. A tendency was observed in favor of the ORG with extra 0.8 kg/d compared to CON cows (25.7 ± 0.43 vs. 24.9 ± 0.44 kg/d; *P* = 0.13). No interaction was observed between treatment group and parity (*P* = 0.52).

### Metabolic status

**Fig. 3:**
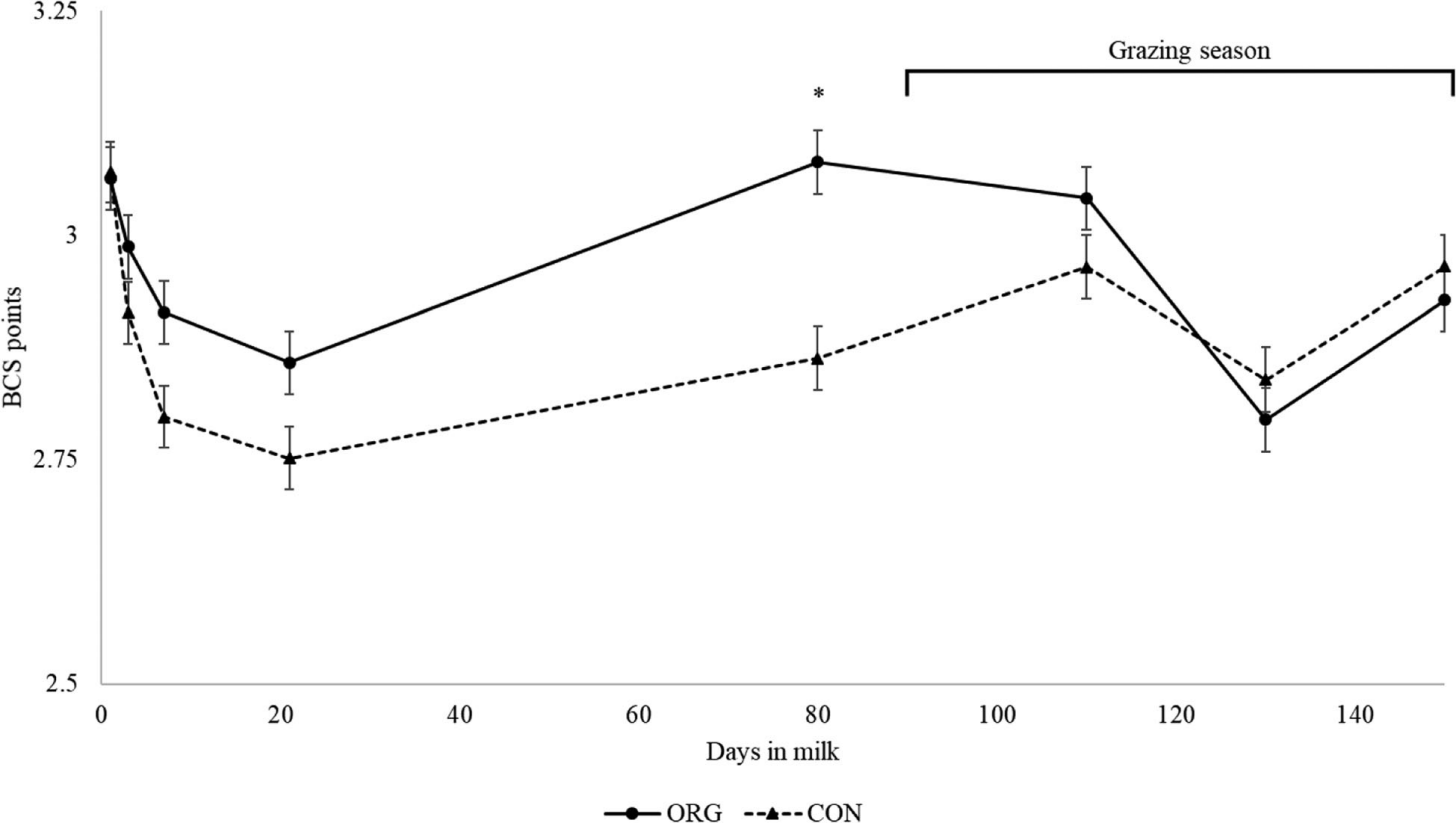
Body condition score fluctuations by treatment groups during the study period. Legend: ORG: cows supplemented with organic rumen protected fat, dot and solid line. CON: cows supplemented with control pellet, triangle and dashed line. Sampling points at 1, 3, 7, 21, 80, 110, 130 and 150 days in milk. Grazing season started after 80 days in milk. Vertical lines show standard errors. *P*-value: * < 0.05

Metabolic status was assessed through the variation of body condition scores during transition and grazing season, and through serum glucose and NEFA determination. Body condition scores before the start of the supplementation (1 DIM) did not show differences between ORG and CON (3.06 ± 0.04 vs. 3.07 ± 0.03 BCS points; *P* = 0.99). Fluctuations of BCS across the study period are presented in Table 2 and Fig. 3. In the multiple comparison mixed model, there was not treatment effect in BCS in the assessment performed at 3, 7, and 21 DIM. However, before the start of grazing around 90 DIM, the ORG group had greater BCS compared to the CON group (3.08 ± 0.04 vs. 2.86 ± 0.04 BCS points; *P* = 0.002). On the other hand, no differences in BCS were observed during grazing or at the end of the supplementation.

**Table 2:** Body condition score (BCS) comparison between rumen protected fat (ORG) and control (CON) groups

Days in milk	Overall				Multiparous				Primiparous			
	ORG	CON	Difference	*P*-value	ORG	CON	Difference	*P*-value	ORG	CON	Difference	*P*-value
1	3.06	3.07	−0.01	0.99	3.05	3.18	−0.13	0.84	3.07	2.97	0.1	0.99
3	2.98	2.91	0.07	0.98	2.99	2.98	0.01	0.99	2.98	2.84	0.14	0.99
7	2.91	2.8	0.11	0.55	2.92	2.93	−0.01	0.99	2.9	2.67	0.23	0.56
21	2.86	2.75	0.11	0.72	2.89	2.88	0.01	0.99	2.83	2.62	0.21	0.81
90a	3.08	2.86	0.22	0.002	2.97	2.87	0.1	0.98	3.2	2.86	0.34	0.04
110b	3.04	2.96	0.08	0.98	3	2.98	0.02	0.99	3.09	2.95	0.14	0.99
130b	2.79	2.84	−0.05	0.99	2.71	2.87	−0.16	0.99	2.88	2.81	0.07	0.99
150b	2.93	2.97	−0.04	0.99	2.89	2.93	−0.04	0.99	2.97	3	−0.03	0.99

Overall BCS least square means from the treatment main effect. Multiparous and primiparous BCS least square means from the interaction between treatment effect and parity. ^a^BCS assessment 7 d before grazing; ^b^BCS evaluated during grazing season

**Fig. 4:**
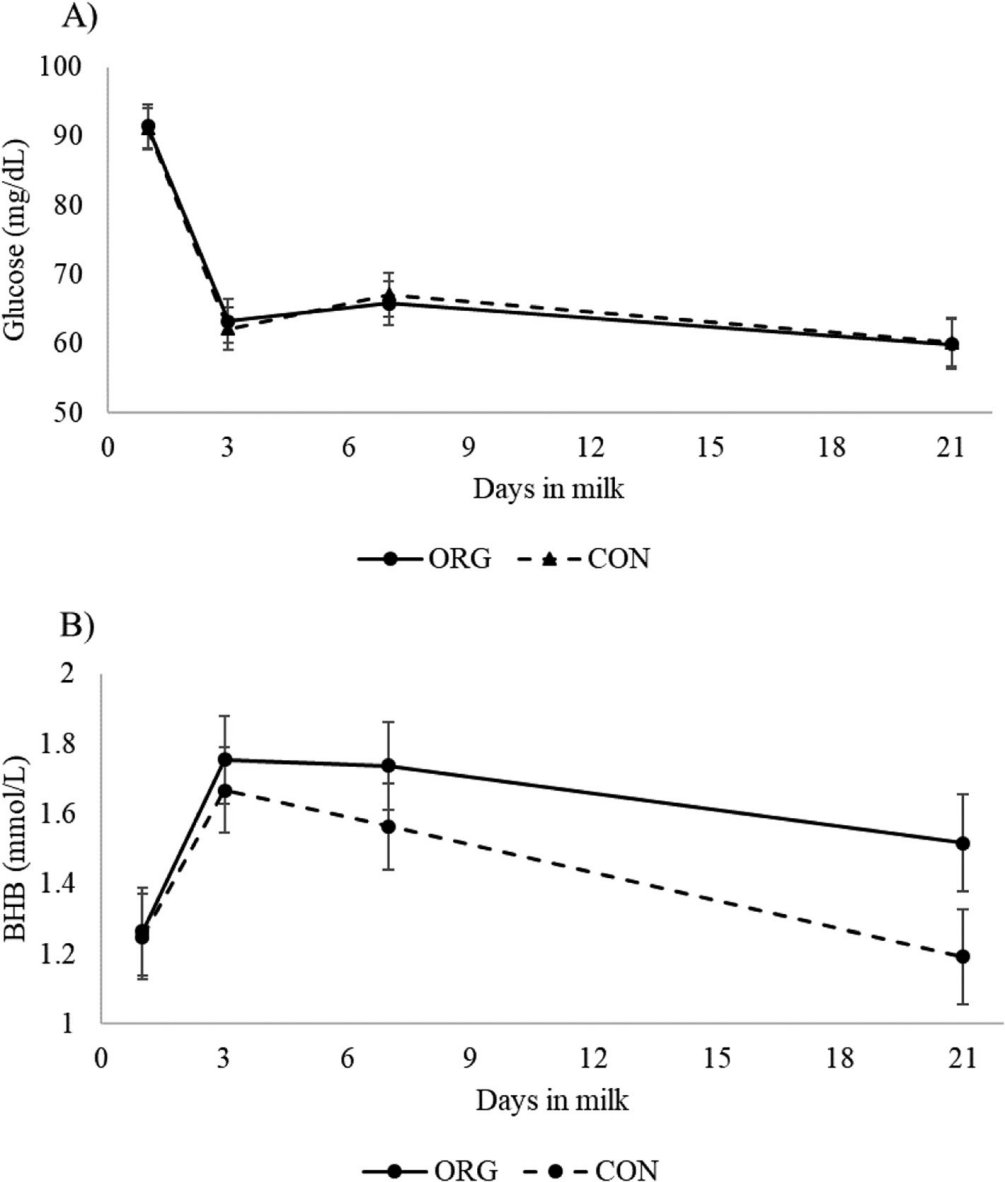
Glucose (**a**), and NEFA (**b**) concentrations from rumen protected fat (ORG) and control (CON) groups. Legend: Least square means and error bars of the serum concentrations of glucose (**a**), and non-esterified fatty acids (NEFA, **b**) at 1, 3, 7 and 21 DIM

Serum concentrations of glucose (mg/dL) and NEFA (mEq/L) were measured at 1, 3, 7 and 21 DIM. One-hundred and forty-seven animals were screened (ORG, *n* = 71; CON, *n* = 76). Serum concentration dynamics throughout the first 21 DIM are presented in in Fig. 4. There was no significant effect of treatment group nor interaction between treatment group and sampling point in glucose, and NEFA concentrations.

### Reproductive performance

There was no association between the resumption of ovarian cyclicity after calving and the supplementation of RPF (*P* = 0.5). Accordingly, the time-to-event analysis showed no differences between treatment groups (*P* = 0.85), where the Kaplan-Meier median time of resumption of ovarian cyclicity was 38 d for ORG (37–48 d) and CON (37–49 d). No differences between treatment groups were established for the time of AI (*P* = 0.5); ORG cows had a Kaplan-Meier median AI time of 67 d (63–76 d) whereas CON cows had a time of 72 d (64–83 d). The number of cows that resulted pregnant from the first AI did not differ between treatment groups (*P* = 0.43). Seventy-seven animals resulted pregnant at 150 DIM. However, there were no differences between treatment groups and parity according to the logistic regression models (*P* = 0.4 and *P* = 0.9, respectively).

### Health outcomes

Fifty-six cows (ORG; *n* = 26, CON; *n* = 30; *P* = 0.6) were diagnosed with health disorders at 150 DIM. There was no association between treatment and disease occurrence (*P* = 0.8). No effect in disease occurrence was observed for the interaction between the variables treatment and parity (*P* = 0.5). Additionally, the occurrence of disease until 305 DIM was evaluated to assess a carry-over effect of the ORG supplementation. Sixty-four animals (ORG; *n* = 30, CON; *n* = 34; *P* = 0.6) were determined sick throughout this period. No effect was found for treatment (ORG vs. CON; *P* = 0.8), parity (*P* = 0.8), or their interaction (*P* = 0.5). The time-to-disease diagnosis for ORG and CON cows was not different within 150 DIM (*P* = 0.6) and 305 DIM (*P* = 0.6).

Voluntary culling was compared during the supplementation period and until 305 DIM. Twenty-three animals (ORG; *n* = 9, CON; *n* = 14; *P* = 0.3) left the herd within 150 DIM. No association was observed between treatment groups and culling (*P* = 0.25). After 305 DIM of follow-up, a total of 44 animals (ORG; *n* = 18; CON; *n* = 26, *P* = 0.2) were culled. No association was found between treatment groups and culling (*P* = 0.16) during this period. On the other hand, the main effect of parity was associated with culling, where multiparous (MP) cows had 3.7 (1.4–9.9; *P* < 0.01) times greater odds of being culled compared to primiparous (PP) cows.

### Measurement of eating time

Parity (*P* = 0.85) and the interaction between treatment group and parity (*P* = 0.17) were not significantly associated with eating time within the time of pellet delivery and therefore were removed from the model. Thus, the final model considered treatment effect, week of supplementation, and their interactions. There was a significant overall effect of treatment on eating time (LSM) by week during the supplementation period (ORG: 17.6 ± 0.4 vs. 16.2 ± 0.4 min/h; *P* = 0.014). However, the multiple comparison test did not indicate significant differences on eating time between treatment groups for specific time points during the supplementation period. Similarly, a significant overall treatment effect was found on daily eating time (ORG: 19.1 ± 0.4 vs. 18 ± 0.4 min/h; *P* = 0.04), but no differences were established when specific days were compared.

## Discussion

The national organic program in the US requires for certified organic dairies a minimum grazing period of not less than 120 days per year, where 30% of the DMI must come from pasture [25]. This characteristic of organic practice poses an extra challenge for dairy cows due to ruminal adaptation to lower energetic dense diets during grazing. Therefore, the addition of tested and organic feed supplement would help to prepare lactating cows in terms of productivity and body condition before grazing avoiding negative effects in productive performance, reproductive goals and health in organic dairies.

Dairy cows have adaptive responses after calving to satisfy the increasing glucose requirements for lactation. The main adaptation against NEB consists in shifting to a lipogenic metabolism, where ketone bodies and free FA are the main source of energy [26]. Therefore, increased levels of β-Hydroxybutyrate (BHB) and NEFA, besides decreased insulin sensitivity might be expected in post-partum dairy cows. However, poor DMI and insufficient supply of glucose precursors triggers fat and muscular tissue mobilization and excessive accumulation of ketone bodies and NEFA, favoring a pathologic state of hyperketonemia [26, 27]. The addition of RPF in rations of lactating dairy cows aims at increasing the energy input during early post-partum. Lipogenic diets are recognized to increase peripheral NEFA and BHB to be used as primary source of energy and to reduce serum glucose [3, 20, 21, 28, 29]. This effect may be explained because lipogenic precursors elicit a surplus of lipid metabolites to be used as energy source [28] and in the β-oxidation of FA [24]. In contrast with this evidence, we did not observe statistical differences in NEFA due to the addition of the organic RPF. Regarding NEFA concentrations, this study population seems to have lower NEFA levels compared to some studies that have evaluated the role of NEB on post-partum diseases [30, 31]. However, a comparison of NEFA concentration should consider the productive system to which cows are subject. In particular, organic dairy cows have lower overall milk production and a higher variation of milk yield between individuals [32]. In the same idea, NEFA concentrations in the study cows resembles that of cows identified as low producing individuals in some reports [33, 34]. However, our study presents limitations regarding a comprehensive assessment of the effect of organic RPF on the metabolic status of the study animals because we do not report data about BHB concentrations that would help to a better understanding of their energy status throughout this trial.

Although carbohydrate pathways are not as well investigated as lipid metabolism in cattle, they also play an important role in the energetic balance. In ruminants, the carbohydrate metabolism is characterized by low circulating levels of glucose, with a high demand by the mammary gland during lactation (0.4 mol/kg of milk) that conditions the level of milk production in dairy cows [21]. Some studies have compared serum glucose in cows under RPF supplementation reporting a tendency of lower serum glucose concentrations among supplemented cows [20, 21]. Accordingly, differences in glucose concentrations between our treatment groups were not observed across sampling points. In order to test the overall effect of the addition of RPF, multiple outcomes that might reflect the cow metabolic status, such as body reserves mobilization, health, production and reproduction, should be considered.

Body condition changes during transition have been associated with milk yield, post-partum health, and decreased fertility [35–37]. Few studies on RPF supplementation have included BCS as a response variable. In this study, BCS was evaluated as a measure of lipid and protein tissue mobilization in response to an expected greater availability of FA absorbed by the small intestine. Before the beginning of supplementation with the organic RPF, the study cows were BC scored within 24 h post-partum not showing significant differences (Table 2). Interestingly, after 90 days of supplementation, ORG cows had lower body condition loss accompanied with greater production during early lactation (Figs. 2 and 3), which could represent and advantage of using organic RPF for pre-grazing conditioning. Contrary to our results, Pappritz et al. in 2011 [29] evaluated BCS between weeks 2–7 of lactation in 30 cows supplemented with RPF conjugated linoleic acid (CLA) and did not find statistical differences, although, this experiment differs to our study in that CLA was the only FA supplemented.

After 30 d in grazing, the group difference in BCS observed at 80 DIM was lost because ORG cows decreased their BCS. Despite this reduction, ORG group sustained higher milk production during the grazing season and the BCS was not different during grazing and by the end of the supplementation (Figs. 2 and 3).

In this study, the addition of RPF increased milk production by 1.6 kg/d during the supplementation period. However, other studies have shown contradictory results on milk yield when RPF was supplemented. McNamara et al. in 2003 [22] tested the differences on milk yield after the supplementation of two commercial RPF for 134 d, using a similar dose to that used in this study (0.45 kg/d). One RPF (Megalac Plus®) increased milk yield by 1.5 kg/d up to 12 weeks of lactation compared to the control group, whereas the no differences were detected for the other RPF (Megapro Gold®). Additionally, Hammon et al. in 2008 [20] determined that cows fed with RPF after a corn starch diet produced 1.8 kg/d more milk compared to control at mid-lactation stage from 80 to 110 DIM. These results agree with what is depicted in the plot showed in Fig. 1, where daily milk yield started to separate gradually between groups and remained different until the end of the supplementation (150 DIM). On the other hand, other studies have not detected improvements on milk yield when RPF was supplemented. For example, Lohrenz et al. in 2010 [21] investigated the inclusion of RPF (*N* = 18) in mid lactation cows (98 DIM) for 4 weeks. Under those study settings, the researchers did not find differences in daily and weekly milk yield, with both groups producing approximately 32.7 kg/day. These results contrast with those found in our study during the mid-lactation stage. Although, the management differs due to grazing and organic production, our study determined that ORG cows produced more milk after 90 DIM (Fig. 2). Another study supplemented post-partum cows (*N* = 14) with RPF tuna oil during the grazing season [38]. Although, the objective of that study was to investigate the effect of tuna oils on sensory characteristics of milk, the extra energy provided by the RPF did not increase milk yield. Contrasting both the published evidence and our results, it seems that RPF supplementation should be maintained during early to middle lactation to affect milk production, since short supplementation studies have not showed significant increments in daily milk yield.

As with milk yield, there are inconsistent results in milk components across published studies on RPF and FA supplementation and the effects of these dietary energy sources are still poorly understood. Contradictory results might be explained by different study settings, sample sizes, intake of the treatment diets, and productive potential of the animals. In this study, we observed consistent increases in daily milk weights during almost all the stages of lactation. However, the next questions that arise are how the energy source provided by the organic RPF was used to overcome NEB and whether the FA in the pellets improved the glucose availability for the mammary gland instead of being used for maintenance. The addition of RPF pellet increased the net energy of lactation, maintenance, and gain of the provided TMR (Table 3), which might explain greater milk production and BCS at mid lactation.

**Table 3:** Ingredients and chemistry composition of the treatment pellets, and total mixed ration

Composition	Supplement pellet		Organilac 200	TMR
	Treatment	Control		
Ingredients, % of DM				
Dehydrated ground alfalfa	20	20	–	–
Rumen protected fat	30	–	93.6	–
Ground corn	40	70	–	–
Molasses	5	5	–	–
Nonfat powder milk	5	5	–	–
Corn silage	–	–	–	16
Hay	–	–	–	41.2
Ray ranch grass	–	–	–	3.3
Farm grain mix	–	–	–	33
Cottonseed	–	–	–	6.2
Chemical composition, % of DM				
DM, %	90.3	85.6	93.6	14.8
CP	9.3	13.4	0.7	14.8
Soluble Protein, % of CP	14.1	13.4	0.2	5.5
ADF protein, % of CP	7.5	0.5	0.34	1.22
NDF protein, % of CP	10.2	1.72	0.54	2.51
ADF	7.9	10	1.2	25.3
NDF	12.8	20	1.9	34.8
Lignin	2.98	2.41	0.44	5.6
Starch	–	–	–	22.6
Crude Fat	28.6	5.1	87.5	4.59
NE Lactation, Mcal/lb. of DM	1.26	0.84	2.36	0.72
NE Maintenance, Mcal/lf of DM	1.3	0.88	2.49	0.73
NE Gain, Mcal/lb. of DM	0.94	0.58	1.86	0.45
Ca	3.84	0.87	9.63	0.85
P	0.26	0.33	0	0.3
Mg	0.17	0.2	0.09	0.3
K	0.69	0.94	0.69	1.47
Na	0.09	0.08	0.03	–
Fe, PPM	204	124	269	–
Mn, PPM	32	23	21	–
Zn, PPM	29	25	6	–
Cu, PPM	9	6	3	–

Improved energy status affects the mammary gland metabolism [20]. Thus, changes in lactose, milk protein and fat have been reported when supplementing RPF [3, 20, 21]. Changes in milk fat may be affected in a greater extent by dietary interventions compared to the protein content, which is putative to the genetic component of the cow with genetic covariances between 33 to 79% [39]. Hammon et al. in 2008 [20] observed that cows supplemented with RPF tended to decrease milk fat. However, other studies agree with our results. For example, McNamara et al. in 2003 [22] did not observe differences in milk fat using supplementing amounts similar to those used in our study. Rumen-protected fats from different sources have also been evaluated regarding milk components. Soybean and tuna oil RPF have shown no differences in milk fat after supplementation [21, 29, 38]. However, Duske et al. in 2009 [3] suggested that differences in milk fat should be observed on the milk FA profiles, especially in unsaturated FA (Palmitoleic acid) that tend to increase with the use of RPF.

In our study milk protein percentage had a constant pattern across treatment groups and evaluation dates. Most reports have concluded that RPF did not alter milk protein percentage [3, 20, 21, 38]. Conversely, McNamara et al. in 2003 [22] concluded that supplementation of commercial RPF reduced milk protein.

FCM is used as a measure of dietary energy and efficiency of dairy systems, which is of interest for dairy farmers [40]. No difference in overall 3.5% FCM between treatment groups was determined in our study. Few studies on RPF supplementation have analyzed FCM. Among those, Hammon et al. in 2008 [20] and Lohrenz et al. in 2010 [21] agreed with our findings, where the inclusion of RPF did not affect FCM.

The main effect of the organic RPF tested in this study was an increase in milk yield and a reduction in body condition loss around 80 DIM. However, other factors that may affect milk yield should be controlled. We made efforts in reducing selection bias by blocking and randomizing the study animals according to their parity (*P* = 0.7), previous lactation productivity (*P* = 0.22), and measuring eating time. Nonetheless, other issues during the implementation of the trial may have affected the ability to accurately attribute an effect to the organic RPF. One factor to consider is the number of cows with dry quarters that by chance might be unbalanced in the treatment groups affecting milk yield of one treatment group. As in organic dairy farming the use of antimicrobial therapy for mastitis is prohibited, a practice to control intramammary infection is to dry severely affected quarters. For this reason, a retrospective analysis was performed to examine whether there was an unbalanced proportion of cows with dry quarters between the two groups and whether there was an interaction between dry quarter proportion per treatment group and milk yield at 150 DIM. The proportion of cows with dry quarters did not differ between ORG and CON groups (19% vs. 14%; *P* = 0.49). To investigate the confounding effect of dry quarters on the average of the daily milk yield up to 150 DIM, a mixed model was used, including treatment group, parity, presence of dry quarters (as binary variable), and the interaction between treatment group, parity, treatment group and dry quarter. The presence of dry quarters and parity interacted with treatment group (*P* < 0.0001 and *P* = 0.002, respectively). Interestingly, cows with dry quarters seem to compensate their milk production and produce more milk in comparison to cows with four functional quarters (33.0 ± 0.14 vs. 31.5 ± 0.06 kg/d; *P* < 0.0001). These differences were also observed when comparing the effect of the inclusion or the organic RPF. Cows in ORG with dry quarters produced 1.8 kg more compared to CON cows with all functional quarters at 150 DIM (32.6 ± 0.18 vs. 31.1 ± 0.1 kg/d; *P* < 0.0001). On the other hand, when comparing treatment groups affected by dry quarters the effect of ORG was diluted by the milk increase compensation in both treatment groups. Therefore, the ORG group with dry quarters produced 32.9 ± 0.18 kg/d at 150 DIM, whereas the CON group with dry quarters produced 33.1 ± 0.3 kg/d (*P* = 0.25).

Additionally, the milk yield analysis was partitioned to consider the grazing season. During this period, ORG cows showed better productive performance, which suggests that RPF can assist in the adaptation to grazing season where most cows lose body condition (Figs. 1 and 2).

The effects of dietary interventions during transition are complex and multifactorial [41]. Several studies have investigated the effect of nutritional interventions during transition on pregnancy proportions, resumption of cyclicity, calving interval, and number of AI per pregnancy, as measures of reproductive performance. However, it is complex to attain greater reproductive efficiency through a single nutritional management, as most strategies are focused into increasing the energy and nutrient availability but their interaction with physiological pathways is not well understood and the outcomes are limited to binary responses. Nonetheless, it has been recognized that some nutrients improve reproductive performance. Rodney et al. in 2018 [41] suggested that increased FA, starch, and metabolizable energy balance intake was positively associated with the proportion of pregnant cows. On the other hand, the authors concluded that the increased intake of rapidly fermentable sugars and high milk protein yield are associated with reduced proportion of pregnant cows. Unfortunately, discrepancies in the study designs and small sample sizes when analyzing binary outcomes limit the validity of the conclusions about the effect of nutritional interventions in dairy cattle [1].

Negative energy balance in dairy cows is associated with reductions on luteinizing hormone pulse frequency, growth rate and diameter of dominant follicle, weight of the corpus luteum, estradiol, and progesterone [28, 42]. Besides the increment of energy density, polyunsaturated FA influence fertility in farm animals by modulating the biosynthesis of prostaglandins, steroids, and the transcriptional regulation of genes involved in the control of fertility [35, 36]. The FA content of the organic RPF used in this study was formulated to match the FA profile of the RPF available in the US market. Consequently, similar effects regarding reproductive performance could be expected.

Overall, there was no significant improvement on the reproductive responses evaluated in this study. Few studies on RPF have investigated reproductive performance. McNamara et al. in 2003 [22] reported no differences in the conception rate at the first AI in dairy cows supplemented with conventional RPF compared to CON cows.

**Fig. 5:**
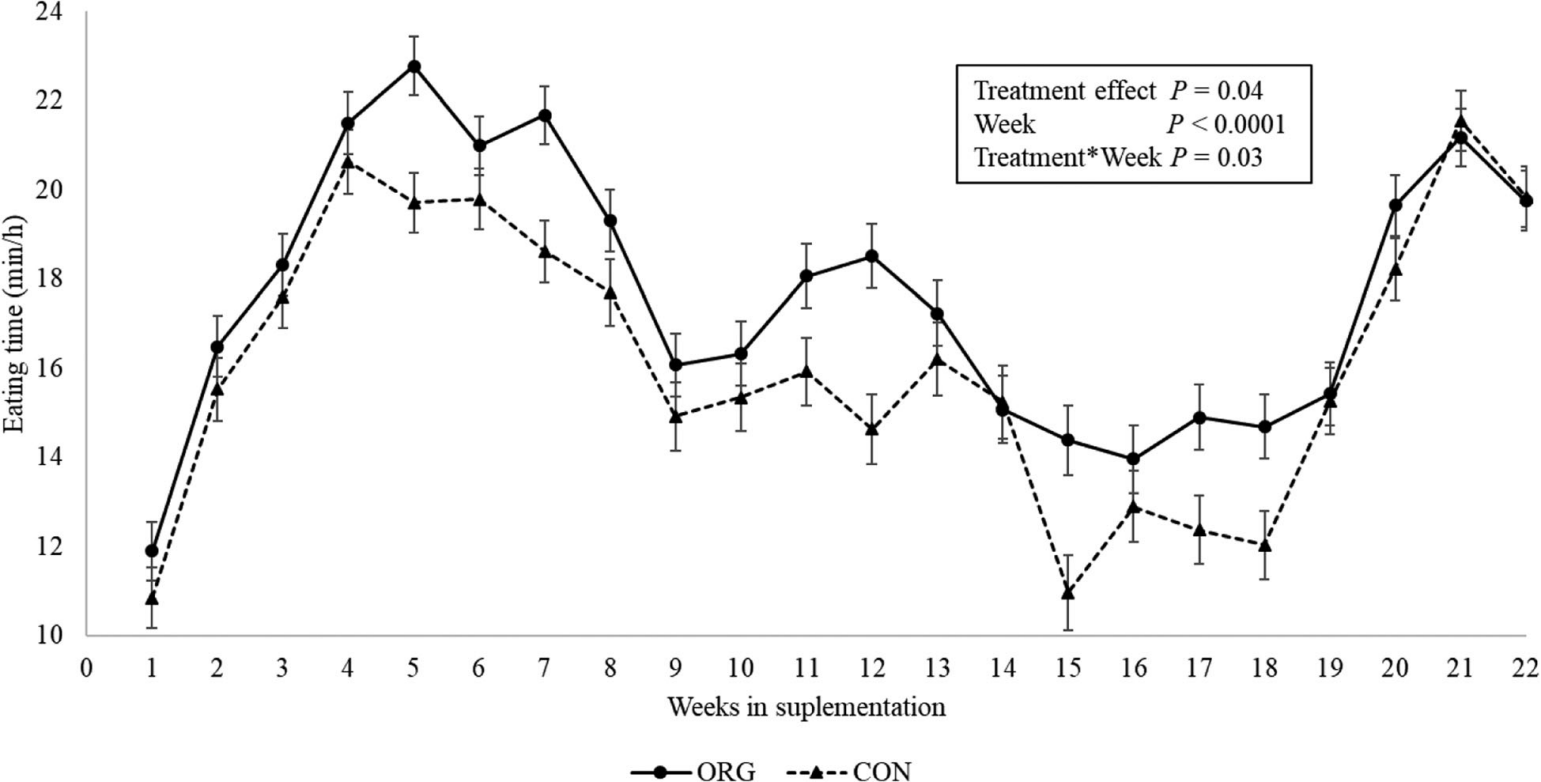
Weekly eating time during the supplementation of the treatment pellets (0700 to 0800 h). Legend: Least square means comparison of the eating time (min/h) for the rumen protected fat (ORG) and control (CON) groups. Eating time was estimated using the Cowmanager® ear tags. The Tukey-Kramer multiple comparison test showed not significant differences for eating time between treatment groups during the same weeks of supplementation

**Fig. 6:**
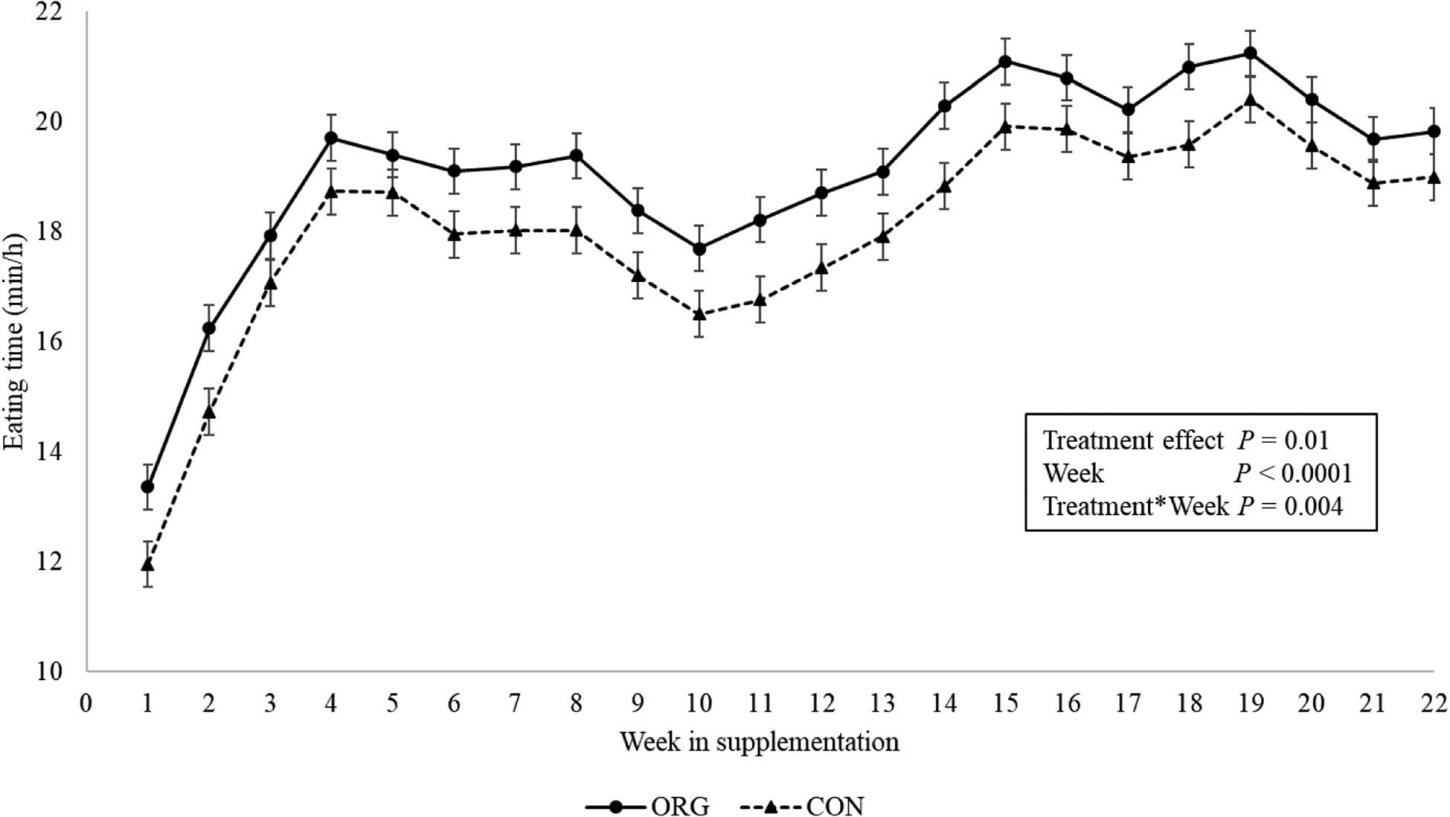
Daily eating time by week during the supplementation period. Legend: Least square means comparison of the eating time (min/h) for the rumen protected fat (ORG) and control (CON) groups. Eating time was estimated using the Cowmanager® ear tags. The Tukey-Kramer multiple comparison test showed not significant differences for eating time between treatment groups during the same weeks of supplementation

Fat supplementation is recognized to affect DMI [2, 22]. When RPF are supplemented to transition dairy cows, DMI has been found to slightly decrease [3, 20, 21]. As our study was performed in a commercial dairy, we were unable to daily assess the TMR consumption by treatment groups, as research subjects were within the same pen, separated only once a day to receive the treatment pellets. Moreover, separating unconsumed TMR per group was unfeasible due to interference with the normal operation of this farm. The differences observed in milk yield and BCS after the supplementation of the organic RPF evaluated in this study can be attributed to the treatment only if extraneous variables that may confound the associations between the treatment effect and the evaluated outcomes are controlled. In this sense, one of the main variables that could bias these results is DMI of the treatment diets by the experimental units. In the idea of measuring and controlling for DMI, we measured eating time using an ear-tag accelerometer sensor. These devices are becoming more common and research studies have validated their use to accurately estimate rumination, eating time, and activity. Research indicates concordance correlation coefficients between 0.7–0.99 when contrasted with visual assessment of eating time [37, 43] and these devices could represent an opportunity when traditional DMI measurement is not feasible. In our study, according to the weekly eating time evaluation, the eating when the treatment pellets were fed, or during the rest of the day did not differ between ORG and CON groups in the weekly evaluations (Figs. 5 and 6).

## Conclusions

This study indicates that supplementation of 0.45 kg/d/head of organic rumen-protected fat increased daily milk yield up to 150 DIM and tended to favor greater milk production up to 305 DIM. Additionally, supplementation reduced the magnitude of body condition loss during at 90 DIM. The inclusion of the tested supplement did not change milk fat and protein, serum glucose and NEFA, reproductive performance, or eating time. The evidence presented in this study suggests that the energy density granted by the organic rumen-protected fat was devoted for milk production and maintenance of body condition and it could be used in organic herds for improvement of such responses.

## Methods

### Study design, animals and management

A randomized blocked controlled trial was conducted from January to July 2017 in a commercial organic certified dairy farm located in Northern Colorado, USA. The sampling frame considered a list of 800 cows in the pre-partum groups within 21 to 15 d before the expected calving date. From these sampling frame, two-hundred and two pregnant non-lactating Holstein cows were randomly selected to conform two study groups for supplementation of an organic RPF pellet (ORG group) or a control pellet (CON group). Both study groups were blocked by party (primiparous [PP] and multiparous [MP] ≥2 lactations), and randomly assigned into the two study groups. Each study group was conformed by 30% of PP cows. The ORG group was supplemented with 1.5 kg/d of a treatment pellet formulated to contain 0.45 kg of the organic RPF (Organilac®, Organic Animal Nutrition, Boulder, CO, USA), whereas the CON group was supplemented with 1.05 Kg of a control pellet formulated to match all feed components except the organic RPF (Table 3). Both treatment pellets were elaborated by Ranch-Way Feeds (Fort Collins, CO, USA).

A research pen housed all the study animals during the trial. The dimensions of the research pen were 47.3 m × 156 m. This pen provided 220 free-stalls, sand bedding, headlocks, outdoor patio and access to ad-libitum water. Both study groups always shared the same facilities, TMR, milking times (07:00, 15:00 and 23:00 h) and management except when the treatment pellets were fed. For pellet delivery, the ORG and CON pellets were individually fed once a day during the trial, after the 07:00 h milking. As individual feeding was required, the study cows were separated in two groups when exiting the milking parlor, based on color marks in both id-ear tags and collars in the control group. After the group separation, the treatment pellets were delivered on top of the TMR. Once the study cows consumed all pellets they were released, and the two groups were allowed to mix in the research pen. More details about sorting methods and efficiency can be found in Manriquez et al. [44].

During the grazing season, the study cows obtained at least 30% of their DMI from pasture. The TMR was based on corn silage (5 to 7%), wheat silage (17 to 19%), grain mix containing soy bean, soy hulls, corn, wheat, and minerals and vitamins (38 to 41%), sorghum silage (5 to 7%), alfalfa hay (2%), grass hay (0 to 1.5%), and pasture grazing (estimates as 30 to 38%). Grazing management considered rotational grazing in pastures based on perennial forages, alfalfa, Italian rye grass, oat rye grass, and teff grass.

### Blood sampling and measurement of blood metabolites

Blood samples were collected from the coccygeal vein within 24 h after calving and at 3, 7, and 21 DIM for determination of glucose, and NEFA concentrations. Blood sample collection was performed after the morning milking (0700 h). One-hundred forty-eight serum samples were subject to laboratory analysis (ORG; *n* = 72; CON; *n* = 76). Vein puncture was performed using vacutainer system tubes without anticoagulant (BD Vacutainer, Franklin Lakes, NJ). After collection, blood was allowed to clot for 1 h at 4 °C, and then centrifuged at 2800 rpm for 15 min. Supernatant was recovered and stored at -20 °C until lab analysis. Glucose (mg/dL) was measured using a handheld meter (FreeStyle Optimum, Abbot Diabetes Care Ltd., Witney, UK) as referenced by Voyvoda and Erdogan in 2010 [45], showing a sensitivity and specificity of 85 and 94%. Non-esterified fatty acid (mEq/L) concentration was determined using a colorimetric enzymatic assay (NEFA-HR-2, Wako Chemicals, Richmond, VA). This assay consisted in the preparations of the provided color reagents and five standards (NEFA concentrations 0,125, 500, and 1000 uEq/L). In 96-well flat bottom plate, 4 uL of the negative control, standards and sample were pipetted in duplicates. Next, 225 uL of the color reagent A were added to each well and incubated at 37 °C for 20 min. After incubation, 75 uL of the color reagent B were added to each well and incubated another 20 min at 37 °C. Finally, the absorbance of the plate was read in a microplate reader at 550 nm, and the NEFA concentration was calculated from the standards using linear regression (Synergy HT, Biotek, Winooski, VT).

### Outcomes and data collection

The response variables measured from the study cows included disease occurrence, culling, mortality, BCS, serum concentration of glucose, and NEFA, milk yield (kg/d), milk components, resumption of ovarian cyclicity at 49 DIM, time to the first AI, pregnancy at the first AI, pregnancy within 150 DIM, and time to pregnancy. All these variables were longitudinally measured from ORG and CON groups from calving until the end of the supplementation.

Farm personnel performed daily health monitoring according to farm protocols during the supplementation period and until 305 DIM. The assessed health outcomes included metritis, endometritis, pyometra, subclinical ketosis, digestive disorders (acidosis, displaced abomasum, diarrhea, constipation), respiratory disease, and lameness. Clinical disease information was retrieved from farm records as well as culling and mortality.

Blind body condition scoring was performed at 1, 3, 7, 21, 80 and 150 DIM using the standard scoring chart of 5 point with a 0.25-point scale [46]. Additionally, BCS was assessed 7 d before the start of the grazing season and at 30, 50 and 75 d after grazing.

Milk was evaluated by daily milk yield up to 150 DIM. Individual milk yield (kg/d) was available from the farm’s milking machine software (ALPRO, DeLaval, Tumba, Sweeden). Milk components were analyzed every 2 weeks by an independent laboratory (The Dairy Authority LLC, Greeley, CO). Components included fat, protein and lactose. Fat corrected milk (FCM = 0.4324*milk in lb. + 16.216*fat content) was calculated at every test day. Additionally, fluctuations in milk yield were evaluated before and during the grazing season. This evaluation was standardized by DIM, and the weekly milk yield averages were compared 1 week before grazing and for up to 5 weeks after grazing started.

During the supplementation trial, only AI was performed as breeding procedure based on heat detection. Cyclicity at 49 DIM was assessed through transrectal ultrasonography evaluating the presence of a corpus luteum at 35 DIM and 49 DIM. The number and date of AI were obtained from farm records. Cyclicity and pregnancy were recorded as binary variables, whereas DIM at AI were analyzed as time-to- event data.

Individual and group eating time (min/d) were estimated using accelerometers (CowManager SensOor, Agis Automatisering BV, Harmelen, the Netherlands) tagged in the left ear [43]. The accelerometers are designed to differentiate spatial movements of the ear being associated to eating, rumination, and activity (walking-running) and provided a reliable approximation of the time cows spent eating the treatment diets, as well as their overall daily eating.

### Statistical analysis

Data analysis was performed using SAS software (SAS 9.4, SAS institute Inc., Cary, NC). Descriptive statistics and univariate analysis for parity, DIM, and disease frequency were performed using Chi square test in SAS (PROC FREQ). The effect of RPF on health outcomes was evaluated using logistic regression (PROC LOGISTIC) and survival analysis (PROC LIFETEST). The occurrence of health disorders, culling and mortality were analyzed as a binary response (1 = diagnosis of any health disorder, culling or mortality; and 0 = absence of health disorder or finished the follow-up period) until 150 and 305 DIM. The logistic models included the effect of RPF, parity and their interaction. Time-to-disease diagnosis, culling and mortality were compared between ORG and CON groups until 150 and 305 DIM. Differences between treatment groups was determined by the Wilcoxon test. Serum concentrations of glucose, and NEFA were analyzed using PROC MIXED for repeated measures. The model included the effect of the RPF, sample point and their interaction.

Analyses of milk yield, milk components, and BCS were performed using PROC MIXED for repeated measures. This model included treatment effect, parity, evaluation times and interaction between treatment effect and parity (1; ≥2 lactation), and evaluation times. The evaluation of daily milk yield considered the sum of the three-daily milking. The model included the fixed effects of treatment (ORG and CON), parity, DIM, and the interaction between treatment effect and DIM. Milk yield analyses during grazing included interaction terms between treatment group, parity and week of evaluation, and a triple interaction term between treatment group, parity, and week of evaluation. Milk fat and protein and FCM were compared by treatment group, parity and evaluation date, including the interaction between treatment group and evaluation date. For BCS at 1 DIM treatment effects, parity and their interaction term were included. To compare BCS between ORG and CON cows, the main effect of treatment, type and evaluation time, and their interactions were included in the mixed model.

Reproductive performance outcomes were analyzed through logistic regression (PROC LOGISTIC), including treatment group, parity, and their interaction terms in the model. Additionally, to explore treatment effect on time-to-first AI and pregnancy, survival analysis was performed (PROC LIFETEST). Wilcoxon *P*-values were used to test equality of strata (ORG and CON) of the survival curves.

Differences in weekly eating time between treatment groups were compared using PROC GLIMMIX, by treatment groups, parity, and their interaction. The analysis was stratified between 0700 and 0800 h to compare eating time during the delivery of the treatment pellets. In addition, overall daily eating time was analyzed in weekly period.

Statistical significance was determined at *P*-values < 0.05. Multiple comparison *P*-values were adjusted through the Tukey-Kramer test. Variables with *P*-values ≤0.15 were kept in the models for confounding control.

## Data Availability

Data sets generated from this study are available upon request to the corresponding author.

## Abbreviations

AI: Artificial insemination
BHB: β-Hydroxybutyrate
CON: Control group
DIM: Days in milk
DMI: Dry matter intake
FA: Fatty acids
FCM: Fat corrected milk
LSM: Least squares means
MP: Multiparous cows
NEB: Negative energy balance
NEFA: Non-esterified fatty acids
ORG: Rumen-protected fat group
PP: Primiparous cows
RPF: Rumen-protected fat
SE: Standard error
TMR: Total mixed ration
VFA: Volatile fatty acids
